# Neonatal abstinence syndrome management in California birth hospitals: results of a statewide survey

**DOI:** 10.1038/s41372-019-0568-6

**Published:** 2020-01-07

**Authors:** Lisa Clemans-Cope, Nikhil Holla, Henry C. Lee, Allison Shufei Cong, Robert Castro, Lisa Chyi, Angela Huang, Kimá Joy Taylor, Genevieve M. Kenney

**Affiliations:** 1 The Urban Institute, 500 L’Enfant Plaza SW, Washington, DC 20037 USA; 2 George Washington University, Washington, DC USA; 3 Division of Neonatal & Developmental Medicine, Stanford University, Stanford, CA USA; 4 California Perinatal Quality Care Collaborative (CPQCC), Stanford University, Stanford, CA USA; 5 Division of Neonatal & Developmental Medicine, Stanford University School of Medicine, Stanford, CA USA; 6 Walnut Creek Medical Center, Kaiser Northern California NAS Workgroup Co-chair, Walnut Creek, CA USA; 7 Department of Neonatology, Santa Clara Valley Medical Center, San Jose, CA USA

**Keywords:** Health services, Health policy

## Abstract

**Objective:**

Assess management of neonatal abstinence syndrome (NAS) in California hospitals to identify potential opportunities to expand the use of best practices.

**Study design:**

We fielded an internet-based survey of 37 questions to medical directors or nurse managers at 145 birth hospitals in California.

**Results:**

Seventy-five participants (52%) responded. Most respondents reported having at least one written protocol for managing NAS, but gaps included protocols for pharmacologic management. Newer tools for assessing NAS severity were not commonly used. About half reported usually or always using nonpharmacologic strategies; there is scope for increasing breastfeeding when recommended, skin-to-skin care, and rooming-in.

**Conclusions:**

We found systematic gaps in care for infants with NAS in a sample of California birth hospitals, as well as opportunities to spread best practices. Adoption of new approaches will vary across hospitals. A concerted statewide effort to facilitate such implementation has strong potential to increase access to evidence-based treatment for infants and mothers.

## Introduction

Neonatal abstinence syndrome (NAS), also called neonatal opioid withdrawal syndrome, is the result of in-utero exposure to opioids. Over the past two decades, increased prevalence of opioid use disorder among pregnant women has sharply increased the incidence of NAS in the United States [1–5]. Nationally, the incidence of NAS in 2014 was 14.4 per 1000 live births, up from 2.8 per 1,000 live births in 2004—over a five-fold increase [6]. The reported incidence of NAS in California increased from 4.6 to 8.1 incidences of NAS per 1000 live births from the period 2009 to 2012 to the period 2013–2016, and rates varied more than tenfold across counties in both periods [7].

The uneven distribution of NAS across geographic areas and characteristics, including rurality [2, 3, 8] and limited evidence on optimal care for NAS, have hindered efforts to standardize and improve care quality. Previous studies have shown hospitals’ approaches to identifying, managing, caring for, and treating opioid-exposed newborns vary widely [9, 10]. Variation across hospitals in resources, staffing, workforce, patient acuity, patient populations, and other factors undoubtedly drives some variation in management of NAS. However, gaps in the evidence base are rapidly being addressed, and consensus is building around best practices and treatment strategies [11–15], providing new momentum to reassess hospitals’ current management of NAS. Studies suggest decreased use of pharmacologic treatment and decreased length of birth hospitalizations are associated with written protocols [16, 17]; infant physiologic measures to assess NAS; [18] promotion of maternal-infant bonding interventions such as breastfeeding [19, 20], “rooming-in” (keeping the mother with the infant during the birth hospitalization) [21], and decreased use of the neonatal intensive care unit (NICU) [22]. These new initiatives have been linked to reductions in hospital cost [23, 24]. As the number of opioid-exposed infants has grown in California, so has the urgency of implementing, testing, and promoting new practices and standardization of care for infants with NAS [18, 23, 25, 26].

This study aimed to assess the current management of NAS in California hospitals, from birth to postdischarge follow-up care, in order to identify opportunities to expand access to evidence-based treatment for opioid-exposed infants and their mothers.

## Methods

We fielded an internet-based survey of 37 questions from June 8, 2018, to August 27, 2018, directed to the medical directors, nurse managers, or other similar contacts at 145 birth hospitals in California, including the member hospitals of the California Perinatal Quality Care Collaborative (CPQCC) and to other hospitals thought to have special care nurseries or NICUs, for which contact information (name, email address, and hospital name) was available. We retrieved respondents’ contact information through collaboration with the CPQCC. Participation was voluntary and responses were confidential. Hospitals could skip questions that did not pertain to their hospital setting. Proportions listed indicate percent of overall hospitals responding to individual questions/topic items. The survey collected data on respondent and hospital characteristics, NAS-related policies and protocols, NAS-related infant and dyad care, discharge practices, and barriers to improving care. The survey instrument is available in the online supplement (Appendix 1). We based many questions on previous surveys and research [9, 15, 27–29], and we gathered responses using the Qualtrics online portal [30]. The 2015 American Hospital Association Annual Survey Database provided hospital characteristics (supplemental Table 1). We estimated 95% confidence intervals for proportions [31], estimated p-values for two-sided tests (*α* = 0.05) to compare characteristics of responding and nonresponding hospitals, and used Stata 15 software to analyze the data. The Urban Institute Institutional Review Board approved this project.

## Results

### Characteristics of participating hospitals

Seventy-five participants (52%) responded to the survey. Among hospitals responding to the survey, 69% (*n* *=* 52) answered all questions in the survey. To determine the potential for bias, we examined differences between characteristics of responding and nonresponding hospitals for which American Hospital Association data were available. For those hospitals (*n* *=* 145), we found no statistically significant differences in geography, number of annual births, bed size, and ownership characteristics (Supplementary Table 1). However, sample sizes for each question varied as not all respondents answered each survey question. Table 1 highlights the characteristics of hospitals participating in the study. The volume of births at the respondents’ hospitals varied from less than 1000 births per year (*n* *=* 12 [17%]) to over 4000 births per year (*n* *=* 8 [11%]). Sixty-seven percent of respondents worked in nongovernment, not-for-profit hospitals (*n* *=* 48). Respondents reported NICU levels as: no NICU (*n* = 6 [8%]); level I or II (*n* = 14 [19%]); level III (*n* = 45 [60%]); and level IV (*n* = 10 [13%]). Thus, most participating hospitals had level III NICUs. Most respondents (*n* = 66 [89%]) usually worked in NICUs (Supplementary Table 2). Most respondents were nurse managers (*n* = 25 [33%]) or NICU medical directors (*n* = 23 [31%]).

**Table 1:** California birth hospitals and patient characteristics, 2018.

Category	% distribution	95% CI		# of hospitals
Annual births (*n* **=** 72), American Hospital Association Annual Survey				
<1000	17%	10%	27%	12
1000–1999	32%	22%	43%	23
2000–2999	24%	15%	35%	17
3000–3999	17%	10%	27%	12
4000+	11%	5%	21%	8
Hospital ownership (*n* **=** 72), American Hospital Association Annual Survey				
Government, nonfederal	19%	12%	30%	14
Nongovernment, not-for-profit	67%	55%	76%	48
Investor-owned (for-profit)	14%	8%	24%	10
Respondent-reported NICU level (*n* **=** 75)				
No NICU in hospital	8%	3%	17%	6
Level I or II	19%	11%	29%	14
Level III	60%	49%	70%	45
Level IV	13%	7%	23%	10
Respondent-reported frequency of maternal-fetal opioid-related exposures (*n* **=** 73)				
Often (1 in 10 patients, or more often)	11%	5%	20%	8
Sometimes (between 1 in 10 patients and 1 in 100 patients)	47%	36%	58%	34
Seldom (1 in 100 patients, or less often)	30%	21%	41%	22
Never	3%	0%	10%	2
Unsure	10%	4%	19%	7
Respondent-reported hospital management of NAS (*n* **=** 75)				
Manages all levels of infants observed for or treated for NAS	81%	71%	89%	61
Manages milder cases and transfers severe cases	13%	7%	23%	10
Transfers all NAS cases	1%	0%	8%	1

Sources: “Hospital Care and Emerging Practices for Treatment of Maternal Opioid Addiction, the Mother–Infant Dyad and Neonatal Abstinence Care: A Survey of California Hospitals” fielded June 2018 to August 2018 by the Urban Institute in collaboration with the California Perinatal Quality-Improvement Collaborative and the California Maternal Quality Care Collaborative; 2015 American Hospital Association Annual Survey Database

*n* = sample size. Denominator includes hospitals that have American Hospital Association data, or, for survey responses, all respondents who selected a response in any part of a survey question (e.g., in a multi-item response table). A respondent who selected an answer in one line of the table but left another line blank are treated as “no” (instead of “missing”) for the line or lines for which they did not respond. 75 out of 145 birth hospitals in the sample responded to the survey

*NAS* neonatal abstinence syndrome, *CI* confidence interval

Forty-seven percent of respondents (*n* = 34) reported that between 1 in 10 and 1 in 100 patients experienced maternal-fetal opioid-related exposures, and 30% (*n* = 22) reported that fewer than 1 in 100 patients experienced these exposures. Most respondents (*n* = 61 [81%]) reported hospital management of all infants observed or treated for NAS, regardless of NAS severity. Across respondents, the median number of infants with NAS related to opioid exposures in the past 6 months was 5 (interquartile range 2 to 10, *n* *=* 57), and the median length of hospital stay for infants treated for NAS was 14 days (interquartile range 10 to 21, *n* *=* 51).

### Written protocols, staff training, and infant assessment related to NAS

Table 2 shows that most respondents reported having at least one written protocol related to NAS for management (*n* = 61 [91%]). Two-thirds or more of respondents reported having a written protocol related to NAS for nursing management (*n* = 49 [73%]), nonpharmacologic management (*n* = 46 [69%]), and breastfeeding (*n* = 44 [66%]). Over half of respondents reported having a written protocol for initiating pharmacologic management (*n* = 39 [58%]) and discharge (*n* = 36 [54%]). Less than half reported having a written protocol for dose escalation of pharmacologic management (*n* = 30 [45%]) or weaning of pharmacologic management (*n* = 28 [42%]). Seventy-seven percent of respondents (*n* = 51) reported that the hospital offered staff training related to NAS, most often during a relevant case (*n* = 36 [56%]). Most respondents (*n* = 55 [86%]) reported staff training on standardized NAS scoring tools.

**Table 2:** Protocols and training related to NAS, sample respondents in California Birth Hospitals in 2018.

Category	%	95% CI		# of hospitals
Hospital has at least one written protocol for hospital management of NAS (*n* **=** 67)	91%	81%	96%	61
Types of written management protocols related to NAS (*n* **=** 67)				
Nursing management	73%	61%	82%	49
Nonpharmacologic management	69%	57%	79%	46
Initiation of pharmacologic management	58%	46%	69%	39
Dose escalation of pharmacologic management	45%	33%	57%	30
Weaning of pharmacologic management	42%	31%	54%	28
Breastfeeding	66%	54%	76%	44
Discharge	54%	42%	65%	36
Transfer	15%	8%	26%	10
Hospital has training related to NAS (*n* **=** 66)	77%	66%	86%	51
Timing of staff training related to NAS (*n* **=** 64)				
At orientation	50%	38%	62%	32
During a relevant case	56%	44%	68%	36
Throughout the year as CME credits	9%	4%	19%	6
At meetings or seminars throughout the year	42%	31%	54%	27
Types of staff training related to NAS (*n* **=** 64)				
Care of substance-exposed infants	86%	75%	93%	55
Standardization of NAS scoring or assessment	86%	75%	93%	55
Training on hospital NAS protocols (if any protocols)	56%	44%	68%	36

Source: “Hospital Care and Emerging Practices for Treatment of Maternal Opioid Addiction, the Mother-Infant Dyad and Neonatal Abstinence Care: A Survey of California Hospitals” fielded June 2018 to August 2018 by the Urban Institute in collaboration with the California Perinatal Quality-Improvement Collaborative and the California Maternal Quality Care Collaborative

*n* = sample size. Denominator includes all respondents who selected a response in any part of the question (e.g., in a multi-item response table). A respondent who selected an answer in one line of the table but left another line blank are treated as “no” (instead of “missing“) for the line or lines for which they did not respond

*NAS* neonatal abstinence syndrome, *CI* confidence interval

### Infant NAS assessments

Ninety-six percent of respondents (*n* = 64) reported using a Finnegan scoring tool (i.e. Finnegan NAS tool, Finnegan NAS Scale Short Form, or other modified Finnegan NAS tool) to assess the severity of NAS symptoms (Supplementary Table 2). Finnegan scoring tools measure signs and symptoms of withdrawal by waking the infant every 2 to 4 h to assess items such as excessive high-pitched crying, muscle tone, tremors, skin excoriations, yawning, and sneezing. A small share of respondents reported using another specified scale, including the Eat, Sleep, Console scale; Lipsitz tool; and the Neonatal Narcotic Withdrawal Index tool (*n* = 6 [9%]) and almost half reported using another unspecified clinical exam or assessment (*n* = 24 [44%]), almost always in addition to a Finnegan scoring tool.

### Treating and feeding infants with NAS

As shown in Table 3, 97% of respondents (*n* = 62) reported nonpharmacologic interventions as the first-line therapy for NAS care. Fifty-five percent of respondents (*n* = 36) reported always or usually providing nonpharmacologic management for NAS, and the remainder reported doing so about half the time (*n* *=* 12 [18%]) or seldom or never (*n* = 17 [26%]). About half of respondents (*n* = 33 [49%]) reported always or usually providing pharmacologic management for NAS, 18% (*n* = 12) reported doing so about half the time, and 34% (*n* = 23) reported doing so seldom or never. For infants with nonpharmacologic management for NAS, 57% of respondents (*n* = 36) reported that the infants receiving nonpharmacologic management for NAS always or usually stay with the mother for the infant's entire stay. In contrast, most infants with pharmacologic management for NAS seldom or never stayed with the mother for the duration of their entire hospitalization (*n* = 50 [86%]).

**Table 3:** Hospital management of NAS, sample respondents in California Birth Hospitals in 2018.

Category	% distribution	95% CI		# of hospitals
First-line therapy for NAS care (*n* **=** 64)				
Nonpharmacologic interventions	97%	89%	100%	62
Pharmacologic interventions	2%	0%	9%	1
Other	2%	0%	9%	1
Frequency of nonpharmacologic management for NAS (*n* **=** 65)				
Always or usually	55%	43%	67%	36
About half the time	18%	11%	30%	12
Seldom or never	26%	17%	38%	17
Frequency of staying with mother for the infant's entire stay, among infants with nonpharmacologic management for NAS (*n* **=** 63)				
Always or usually	57%	45%	69%	36
About half the time	8%	3%	18%	5
Seldom or never	35%	24%	47%	22
Frequency of pharmacologic management for NAS (*n* **=** 68)				
Always or usually	49%	37%	60%	33
About half the time	18%	10%	29%	12
Seldom or never	34%	24%	46%	23
Frequency of staying with mother for the infant's entire stay, among infants with pharmacologic management for NAS (*n* **=** 58)				
Always or usually	7%	2%	17%	4
About half the time	7%	2%	17%	4
Seldom or never	86%	75%	93%	50

Source: “Hospital Care and Emerging Practices for Treatment of Maternal Opioid Addiction, the Mother-Infant Dyad and Neonatal Abstinence Care: A Survey of California Hospitals” fielded June 2018 to August 2018 by the Urban Institute in collaboration with the California Perinatal Quality-Improvement Collaborative and the California Maternal Quality Care Collaborative

*n*  = sample size. Denominator includes all respondents who selected a response in any part of the question (e.g., in a multi-item response table). A respondent who selected an answer in one line of the table but left another line blank are treated as “no” (instead of “missing”) for the line or lines for which they did not respond

*NAS* neonatal abstinence syndrome, *CI* confidence interval

As shown in Table 4, the most common nonpharmacologic NAS management interventions related to the environment were swaddling (*n* = 64 [100%]), quiet environment (*n* = 60 [94%]), and low-level lighting (*n* = 55 [86%]). Nonpharmacologic NAS interventions related to the mother-infant dyad were not as common, including skin-to-skin care (*n* = 44 [71%)], breastfeeding (*n* = 37 [60%)], and rooming-in (*n* = 28 [44%)]. Only 33% of respondents (*n* = 16) indicated that rooming-in was offered in the NICU (Supplementary Table 2).

**Table 4:** Nonpharmacologic interventions related to NAS, sample respondents in California Birth Hospitals in 2018.

Routinely provided nonpharmacologic interventions	%	95% CI		# of hospitals
Environment (*n* **=** 64)				
Swaddling	100%	93%	100%	64
Quiet environment	94%	85%	98%	60
Low-level lighting	86%	75%	93%	55
Covered isolette/crib	69%	57%	79%	44
Sleep positioning	69%	57%	79%	44
Warm blanket	39%	28%	51%	25
Music therapy	17%	10%	28%	11
Rooming-in	44%	32%	56%	28
Dyad-specific care (*n* **=** 62)				
Skin-to-skin care	71%	59%	81%	44
Breastfeeding	60%	47%	71%	37
Physical intervention (*n* **=** 62)				
Holding	95%	86%	99%	59
Gentle rocking	81%	69%	89%	50
Gentle containment/pressure	60%	47%	71%	37
Slow infant handling	56%	44%	68%	35
Massage	21%	13%	33%	13
Other (*n* **=** 62)				
Volunteer “cuddler” program	48%	36%	61%	30
Empowering messages to caregiver	21%	13%	33%	13
Delaying circumcision	16%	9%	27%	10
Acupuncture	3%	0%	12%	2

Source: “Hospital Care and Emerging Practices for Treatment of Maternal Opioid Addiction, the Mother-Infant Dyad and Neonatal Abstinence Care: A Survey of California Hospitals” fielded June 2018 to August 2018 by the Urban Institute in collaboration with the California Perinatal Quality-Improvement Collaborative and the California Maternal Quality Care Collaborative

*n* = sample size. Denominator includes all respondents who selected a response in any part of the question (e.g., in a multi-item response table). A respondent who selected an answer in one line of the table but left another line blank are treated as “no” (instead of “missing”) for the line or lines for which they did not respond

*NAS* neonatal abstinence syndrome, *CI* confidence interval

**Fig. 1:**
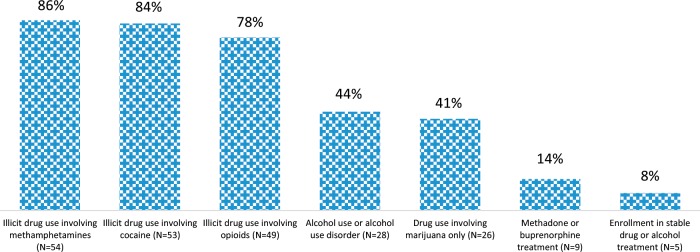
Maternal drug and alcohol use or treatment in which breastfeeding is discouraged, among mothers of infants observed or treated for NAS (*N* = 63), sample respondents in California Birth Hospitals in 2018.

Among all infants, most respondents reported breastfeeding as common practice within the hospital (n = 48 [80%]). Among mothers of infants observed or treated for NAS, breastfeeding is still rarely discouraged for those in methadone or buprenorphine treatment (*n* = 9 [14%]). However, as shown in Fig. 1, breastfeeding is very often discouraged when illicit drug use involves methamphetamines (*n* = 54 [86%]), cocaine (*n* = 53 [84%]), opioids (*n* = 49 [78%]), alcohol use or alcohol use disorder (*n* = 28 [44%]), or marijuana-only drug use (*n* = 26 [41%]).

As shown in Table 5, the most common first-line pharmacologic treatments for NAS were oral morphine (*n* = 43 [73%]) and methadone (*n* = 16 [27%]), and the most common second-line treatments were phenobarbital (*n* = 30 [51%]) and clonidine (*n* = 19 [32%]).

**Table 5:** Most common first- and second-line pharmacologic treatment of NAS, sample respondents in California Birth Hospitals in 2018.

Medication	Most common first-line medication (*n* = 59)				Most common second-line medication (*n* = 59)			
	%	95% CI		# of hospitals	%	95% CI		# of hospitals
Morphine (oral)	73%	60%	83%	43	12%	6%	23%	7
Methadone	27%	17%	40%	16	24%	15%	36%	14
Morphine (IV)	10%	4%	21%	6	14%	7%	25%	8
Phenobarbital	8%	3%	19%	5	51%	38%	63%	30
Clonidine	7%	2%	17%	4	32%	22%	45%	19
Diazepam	0%	0%	7%	0	7%	2%	17%	4
Tincture of opium (e.g., Laudanum, deodorized opium tincture)	0%	0%	7%	0	7%	2%	17%	4
Buprenorphine	0%	0%	7%	0	5%	1%	14%	3
Paregoric (e.g., camphorated tincture of opium)	0%	0%	7%	0	5%	1%	14%	3

Source: “Hospital Care and Emerging Practices for Treatment of Maternal Opioid Addiction, the Mother-Infant Dyad and Neonatal Abstinence Care: A Survey of California Hospitals” fielded June 2018 to August 2018 by the Urban Institute in collaboration with the California Perinatal Quality-Improvement Collaborative and the California Maternal Quality Care Collaborative

*n* = sample size. Denominator includes all respondents who selected a response in any part of the question (e.g., in a multi-item response table). A respondent who selected an answer in one line of the table but left another line blank are treated as “no” (instead of “missing”) for the line or lines for which they did not respond

*NAS* neonatal abstinence syndrome, *CI* confidence interval

### NAS discharge and postdischarge follow-up

Table 6 shows that most respondents reported that infants with NAS that received pharmacologic therapy were seldom or never discharged with at-home pharmacologic treatment (*n* = 46 [81%]). Forty-three percent of respondents (*n* *=* 27) offered home visit nursing programs, and 33% (*n* = 21) offered referral to a specialized program for NAS or high-risk infants. Forty-four percent of respondents (*n* = 28) reported familiarity with home visiting services in their communities.

**Table 6:** Discharge practices and postdischarge follow-up care and knowledge related to NAS, sample respondents in California Birth Hospitals in 2018.

Category	%	95% CI		# of hospitals
Seldom or never discharge infants while still on pharmacologic therapy for NAS (*n* = 57)	81%	68%	89%	46
Postdischarge follow-up provided to the parent, guardian, or caretaker of infants under observation or being treated for NAS related to opioid exposure (*n* = 63)				
Scheduling of pediatrician visits	89%	79%	95%	56
Referral for pediatrician visits	68%	56%	78%	43
Home visit nursing	43%	31%	55%	27
Scheduling of maternal primary care physician visits	37%	26%	49%	23
Referral to a specialized program for NAS or high-risk infants	33%	23%	46%	21
Referral for maternal primary care physician visits	29%	19%	41%	18
Familiar with home visiting services (*n* = 63)	44%	33%	57%	28

Source: “Hospital Care and Emerging Practices for Treatment of Maternal Opioid Addiction, the Mother-Infant Dyad and Neonatal Abstinence Care: A Survey of California Hospitals” fielded June 2018 to August 2018 by the Urban Institute in collaboration with the California Perinatal Quality Improvement Collaborative and the California Maternal Quality Care Collaborative

*n*  = sample size. Denominator includes all respondents who selected a response in any part of the question (e.g., in a multi-item response table). A respondent who selected an answer in one line of the table but left another line blank are treated as “no” (instead of “missing”) for the line or lines for which they did not respond

*NAS* neonatal abstinence syndrome, *CI* confidence interval

### Respondents’ ideas for improving NAS care

**Fig. 2:**
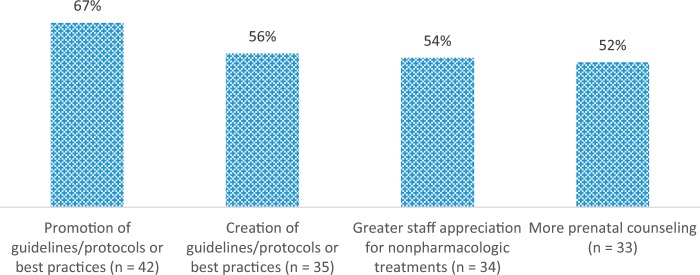
Most frequently cited ideas for improving care for NAS at your hospital (*N* = 63), sample respondents in California Birth Hospitals in 2018.

Respondents were asked to indicate the top three solutions for improving NAS care at their hospital (Fig. 2; Supplementary Table 3). Among the frequently identified solutions were promoting guidelines and best practices (*n* = 42 [67%]), creating guidelines or best practices (*n* = 35 [56%]), and increasing staff appreciation for nonpharmacologic treatments (*n* = 34 [54%]). Almost all respondents wanted to receive additional guidance regarding quality of NAS care, with 59% (*n* = 39) reporting that it would be “very useful,” and 35% (*n* = 23) reporting that it would be “somewhat useful” (data not shown).

## Discussion

Our survey revealed both consistency and variation in NAS management across California hospitals and identified gaps between current practice and recommended best practices as reflected in recent clinical guidance such as the 2012 American Academy of Pediatrics guidelines [15], other expert consensus guidance, and emerging best practice innovation [11–14]. Differences in hospital practices are likely driven in part by regional characteristics, patient demographics and clinical factors, hospital resource challenges, and other factors. Yet the study revealed several promising opportunities to expand the use of best practices.

Overall, 91% of sampled California birth hospitals had at least one NAS management protocol. Seventy-three percent of respondents had NAS protocols on nursing care, 69% had NAS protocols on nonpharmacologic treatment, and 58% had NAS protocols on initiating pharmacologic treatment. Protocols on other aspects of NAS care were less common; for example, only 42% of hospitals have protocols for weaning of pharmacologic treatment of NAS, which has been shown to improve NAS outcomes [16, 32]. Respondents recognized this gap. In fact, creating and promoting guidelines, protocols, or best practices was most commonly cited as the most important solution for improving hospital care for NAS.

Our findings also suggest that newer tools for assessing NAS severity and infant physiologic parameters are not commonly used in California hospitals, despite emerging research and quality-improvement initiatives suggesting that these newer tools may reduce pharmacologic treatment and length of infant hospital stays [18, 33]. In order to improve outcomes, hospitals in California could consider testing newer assessments such as the Eat, Sleep, Console scale and tracking evaluation results that assess health and neurodevelopmental outcomes.

Though almost all respondents reported nonpharmacologic interventions as the first-line therapy for NAS care, only about half of respondents usually or always used these strategies.

Several quality-improvement organizations have worked toward increasing use of nonpharmacologic treatment protocols in birth hospitals, including in NICUs [34, 35]. California has yet to embark on a concerted statewide effort to facilitate such implementation, though recent, newly available federal funding will support a statewide expansion of evidence-based treatment for infants with NAS [36]. Our findings show that there is room for increasing use of nonpharmacologic treatment strategies in the state, including breastfeeding when recommended, skin-to-skin care, and rooming-in. According to current guidelines, maternal substance use is not a categorical contraindication to breastfeeding [37, 38], and mothers enrolled in methadone or buprenorphine maintenance treatment are encouraged to breastfeed regardless of dose, as only a small amount of methadone or buprenorphine passes into breast milk [39], and breastfeeding decreases incidence of NAS and pharmacotherapy [40]. In addition, our findings show that less than two-thirds of hospitals routinely provided skin-to-skin care, and less than half of hospitals routinely provide rooming-in. Boosting these nonpharmacologic interventions is important, as both are associated with lower need for pharmacologic treatment and shorter hospitalizations [18, 21].

Increasing the share of hospitals with written protocols for initiating nonpharmacologic management could decrease the use of pharmacologic treatments to promote parental presence and provision of care as well as standardize and improve the quality of pharmacologic treatments (e.g., initiation, weaning, discontinuation) when necessary [16, 18]. We found substantial variation and room for improvement in pharmacologic treatments; for example, we found that the most common first-line pharmacologic treatments for NAS are oral morphine and methadone, though studies from clinical trials and treatment settings show that buprenorphine may be superior to either of these [41], and research suggests that methadone treatment may be superior to morphine treatment [42]. As results become available from clinical trials comparing pharmacologic treatments for NAS, hospital guidelines and protocols should be updated to maximize treatment strategies that reduce pharmacologic exposure and length of hospital stays.

Increasing the number of hospitals that routinely provide referrals to home visiting services and specialized programs for NAS or other high-risk infants could also improve outcomes for these vulnerable infants. That will likely require enough trained social workers and other staff and resources to be available to link these mothers to the social services they need. It may also require addressing gaps in community resources available to provide appropriate care after discharge.

Most respondents suggested that creating and promoting clinical guidelines, protocols, or best practices are the most important actions for improving NAS hospital care. Stakeholder collaboratives could be engaged in developing guidelines and protocols, which can foster stakeholder buy-in, facilitate consensus, and develop effective strategies to promote adoption and adherence [43, 44].

Adoption of new approaches will not be “one size fits all.” Implementation of new protocols and procedures to improve NAS infant outcomes will be a process, as various facilitators and barriers will influence the implementation approaches that make sense for individual hospitals. First, hospitals that are overwhelmed and understaffed may be more likely to put any suspected NAS infant in the NICU, where the nurse-patient ratio is higher, but nonpharmacologic interventions are often harder to promote. These hospitals could assess whether rooming-in strategies can be successful at current nurse-patient ratios, particularly with fussy and challenging newborns. In addition, addressing concerns around liability and fear of lawsuits may help change practice patterns such as overreliance on NICU and pharmacotherapy. Hospitals may also lack providers with NAS experience or have the time required to provide time-intensive care for these babies. Hospitals may need to include clinical and child welfare staff to implement approaches that rely more on nonpharmacologic interventions.

Second, some hospitals and practice models may more easily train for and adapt to new approaches while others may require more substantial time and investment to change practice culture and attitudes. Hospitals will need to address staff needs for initial and ongoing training, and higher turnover will make it hard to ensure all staff are trained on the new protocols and skills. Some staff may need to develop new skills for coaching women on how to soothe and feed infants with withdrawal symptoms. Some hospitals may need substantial investment to change physician culture and attitudes. Thus, hospitals may be reluctant to engage in these new approaches if all are held to the same expectations and timetable.

Third, hospitals with different levels of NICU/no NICU will have very different resource challenges beyond staffing issues, including bed availability and capital investment to expand spaces for babies with substance exposure. This space may also be needed to support and allow these mothers to sort out social situations and needs such as housing, intimate partner violence, or connection with a substance use treatment provider.

One limitation of the study is that the CPQCC, from which we drew our sample, is geared toward NICUs. Consequently, survey respondents worked primarily in NICUs rather than in well-newborn nurseries; thus, NICU experiences are overrepresented in this study. In addition, unobserved characteristics of responding and nonresponding hospitals could differ in meaningful ways, such as the share of infant opioid exposures relating to methadone, buprenorphine, or prescription opioids compared with the share relating to illicit opioid exposure. Our study was also limited in that not all respondents answered each question. Finally, though we drew content on previous research for developing the survey questions, many of the survey questions have not been tested, which may limit their reliability and validity.

## Conclusions

Our study found potential gaps in care for infants affected by NAS in a sample of California birth hospitals and opportunities to overcome these gaps, with more than half of study respondents self-identifying strategies to improve care. Hospitals could improve care for infants and mothers by developing and promoting protocols for infant care related to NAS, including supporting nonpharmacologic treatment as first-line NAS treatment, by testing replacement of the Finnegan or modified-abstinence scales with other potentially more clinically relevant tools, promoting maternal-infant bonding and breastfeeding, rooming-in, and avoiding the NICU when possible. There is also considerable scope for improving appropriate outpatient care after discharge. While some of the hospitals in our survey that did not have protocols for NAS care may not been as affected by NAS as much as others, mitigating the adverse effects of NAS in California will require that all hospitals proactively implement strategies to ensure that affected infants have the best opportunities for care from birth. Additionally, longer-term health and neurodevelopmental outcomes can be tracked to help establish these strategies as best practice rather than relying on short-term inpatient health outcomes alone.

## Supplementary information

Supplemental tables

Survey Instrument
