# Supplementary material for: Neonatal abstinence syndrome management in California birth hospitals: results of a statewide survey
Source: J Perinatol. 2020 Jan 7;40(3):463–72. doi: 10.1038/s41372-019-0568-6 (PMC7042156; doi:10.1038/s41372-019-0568-6)
Supplement: Supplementary file 1 — Supplemental tables [file 41372_2019_568_MOESM1_ESM.pdf]

**Supplemental Table 1. Characteristics of Responding and Nonresponding California Birth Hospitals Matched with American Hospital Association Annual Survey Data, 2018.**

| Categories | Responding Hospitals Matched with AHA Data (n = 75) | | | | Nonresponding Hospitals Matched with AHA Data (n = 70) | | | | |
|---|---|---|---|---|---|---|---|---|---|
| | % distribution | 95% CI | | Total *n* | % distributio | 95% CI | | Total *n* | P- value* |
| **Geographical setting** | | | | | | | | | 0.348 |
| Metropolitan | 97% | 90% | 100% | 72 | 95% | 87% | 99% | 66 | |
| Micropolitan | 1% | 0% | 8% | 72 | 5% | 1% | 13% | 66 | |
| Rural | 1% | 0% | 8% | 72 | 0% | 0% | 7% | 66 | |
| **Annual births** | | | | | | | | | 0.761 |
| < 1000 | 17% | 10% | 27% | 72 | 12% | 6% | 22% | 66 | |
| 1000-1999 | 32% | 22% | 43% | 72 | 30% | 21% | 42% | 66 | |
| 2000-2999 | 24% | 15% | 35% | 72 | 32% | 22% | 44% | 66 | |
| 3000-3999 | 17% | 10% | 27% | 72 | 18% | 11% | 29% | 66 | |
| 4000+ | 11% | 5% | 21% | 72 | 8% | 3% | 17% | 66 | |
| **Total hospital beds** | | | | | | | | | 0.725 |
| < 100 | 6% | 2% | 14% | 72 | 5% | 1% | 13% | 66 | |
| 100-199 | 21% | 13% | 32% | 72 | 27% | 18% | 39% | 66 | |
| 200-299 | 28% | 19% | 39% | 72 | 23% | 14% | 34% | 66 | |
| 300-399 | 26% | 18% | 38% | 72 | 32% | 22% | 44% | 66 | |
| 400+ | 19% | 12% | 30% | 72 | 14% | 7% | 24% | **66** | |
| **Total obstetric beds** | | | | | | | | | 0.657 |
| < 20 | 34% | 23% | 46% | 62 | 24% | 15% | 37% | 58 | |
| 20-39 | 47% | 35% | 59% | 62 | 50% | 38% | 62% | 58 | |
| 40-59 | 15% | 8% | 26% | 62 | 19% | 11% | 31% | 58 | |
| 60+ | 5% | 1% | 14% | 62 | 7% | 2% | 17% | 58 | |
| **Neonatal intensive care beds** | | | | | | | | | 0.897 |
| < 20 | 58% | 46% | 70% | 62 | 60% | 47% | 72% | 58 | |
| 20-39 | 29% | 19% | 41% | 62 | 28% | 18% | 40% | 58 | |
| 40-59 | 10% | 4% | 20% | 62 | 7% | 2% | 17% | 58 | |
| 60+ | 3% | 0% | 12% | 62 | 5% | 1% | 15% | 58 | |
| **Health care system type** | | | | | | | | | 0.618 |
| Centralized health system | 16% | 8% | 29% | 50 | 20% | 11% | 33% | 51 | |
| Moderately centralized health system | 12% | 5% | 24% | 50 | 8% | 3% | 19% | 51 | |
| Decentralized health system | 52% | 39% | 65% | 50 | 61% | 47% | 73% | 51 | |
| Independent hospital system | 18% | 10% | 31% | 50 | 12% | 5% | 24% | 51 | |
| Not enough data | 2% | 0% | 11% | 50 | 0% | 0% | 8% | 51 | |
| **Hospital ownership** | | | | | | | | | 0.189 |
| Government, nonfederal | 19% | 12% | 30% | 72 | 9% | 4% | 19% | 66 | |
| Nongovernment, not-for-profit | 67% | 55% | 76% | 72 | 79% | 67% | 87% | 66 | |
| Investor-owned (for-profit) | 14% | 8% | 24% | 72 | 12% | 6% | 22% | 66 | |

Source: 2015 American Hospital Association Annual Survey Database.

Notes: NAS = neonatal abstinence syndrome. AHA = American Hospital Association. CI = confidence interval. n = sample size.

* Overall P- values based on Pearson's Chi square test of significance between responding and nonresponding hospitals on categories, for a two-sided test at significance level α = 0.05.

**Supplemental Table 2. Screening, Assessments, and Location of Rooming-In Related to NAS, Sample Respondents in California Birth Hospitals in 2018**

| Interventions | % | 95% CI | | # of hospitals |
|---|---|---|---|---|
| **Location of rooming-in for mothers of infants being observed or treated for NAS (n = 49)** | | | | |
| Well-newborn nursery | 20% | 11% | 34% | 10 |
| NICU | 33% | 21% | 47% | 16 |
| PICU | 2% | 0% | 12% | 1 |
| Pediatrics inpatient unit | 31% | 19% | 45% | 15 |
| Maternity/postpartum unit | 59% | 45% | 72% | 29 |
| **Methods of NAS assessment (n = 67)** | | | | |
| Finnegan NAS tool, Finnegan NAS Scale Short Form, or other modified Finnegan NAS tool | 96% | 87% | 99% | 64 |
| ESC (Eat, Sleep, Console) scale, Lipsitz tool (Narcotic Withdrawal Score), or Neonatal Narcotic Withdrawal Index tool | 9% | 4% | 19% | 6 |
| Other clinical exams or assessments | 44% | 32% | 58% | 24 |

Source: "Hospital Care and Emerging Practices for Treatment of Maternal Opioid Addiction, the Mother-Infant Dyad and Neonatal Abstinence Care: A Survey of California Hospitals" fielded June 2018 to August 2018 by the Urban Institute in collaborative research with the California Perinatal Quality Improvement Collaborative and the California Maternal Quality Care Collaborative.

Notes: NAS = neonatal abstinence syndrome. CI = confidence interval. n = sample size. NICU = neonatal intensive care unit. PICU = pediatric intensive care unit. Denominator includes all respondents who selected a response in any part of the question (e.g., in a multi-item response table). A respondent who selected an answer in one line of the table but left another line blank are treated as “no” (instead of "missing") for the line or lines for which they did not respond.

**Supplemental Table 3. Solutions to Improving Care for NAS, Sample Respondents in California Birth Hospitals in 2018**

| Category | % | 95% CI | | # of hospitals |
|---|---|---|---|---|
| **Solutions to improving care for NAS at your hospital (n = 63)** | | | | |
| Promotion of guidelines/protocols or best practices (n = 42) | 67% | 54% | 77% | 42 |
| Creation of guidelines/protocols or best practices (n = 35) | 56% | 43% | 67% | 35 |
| Greater staff appreciation for nonpharmacologic treatments (n = 34) | 54% | 42% | 66% | 34 |
| More prenatal counseling (n = 33) | 52% | 40% | 64% | 33 |
| More clinician education (n = 24) | 38% | 27% | 50% | 24 |
| More follow-up with infants and parents/caregivers (n = 20) | 32% | 22% | 44% | 20 |
| Greater parents/caregiver appreciation for nonpharmacologic treatments (n = 18) | 29% | 19% | 41% | 18 |
| More patient engagement/interest (n = 17) | 27% | 18% | 39% | 17 |
| Addressing patient stigma related to perinatal substance use (n = 17) | 27% | 18% | 39% | 17 |
| More integration between different types of care and care providers (n = 13) | 21% | 12% | 32% | 13 |
| Addressing clinician stigma related to perinatal substance use (n = 12) | 19% | 11% | 31% | 12 |
| More hospital internal/leadership support (n = 9) | 14% | 7% | 25% | 9 |
| More culturally appropriate care practices (n = 8) | 13% | 6% | 23% | 8 |
| Providing a higher level of care (e.g., a higher-level nursery; n = 4) | 6% | 2% | 16% | 4 |
| More transparency in current practices (n = 4) | 6% | 2% | 16% | 4 |
| Improving payment systems (n = 4) | 6% | 2% | 16% | 4 |
| More clinician engagement/interest (n = 3) | 5% | 1% | 14% | 3 |

Source: "Hospital Care and Emerging Practices for Treatment of Maternal Opioid Addiction, the Mother-Infant Dyad and Neonatal Abstinence Care: A Survey of California Hospitals" fielded June 2018 to August 2018 by the Urban Institute in collaboration with the California Perinatal Quality Improvement Collaborative and the California Maternal Quality Care Collaborative.

Notes: NAS = neonatal abstinence syndrome. CI = confidence interval. n = sample size. Denominator includes all respondents who selected a response in any part of the question (e.g., in a multi-item response table). A respondent who selected an answer in one line of the table but left another line blank are treated as “no” (instead of "missing") for the line or lines for which they did not respond.
