# Supplementary material for: Neonatal abstinence syndrome management in California birth hospitals: results of a statewide survey
Source: J Perinatol. 2020 Jan 7;40(3):463–72. doi: 10.1038/s41372-019-0568-6 (PMC7042156; doi:10.1038/s41372-019-0568-6)
Supplement: Supplementary file 2 — Survey Instrument [file 41372_2019_568_MOESM2_ESM.docx]

#
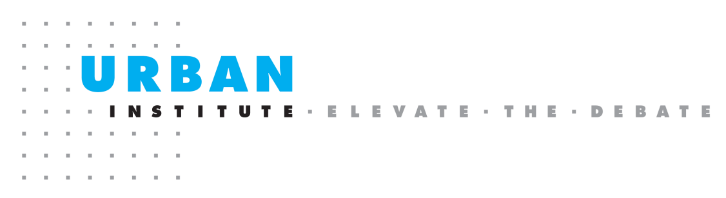
Hospital Care and Emerging Practices for Treatment of Maternal Opioid Addiction, the Mother-Infant Dyad and Neonatal Abstinence Care: A Survey of California Hospitals

Lisa Clemans-Cope and Nikhil Holla (Urban Institute, 2018)

- 1. I am a (select all that apply) [Options randomized]:
     1. Nurse manager
     2. Labor and delivery medical director
     3. NICU medical director
     4. Other, please specify:
  2. During the past 6 months, I worked in (select all that apply) [Options randomized]:
     1. Prenatal Care
     2. Labor and Delivery
     3. Post-partum unit
     4. NICU
     5. PICU
     6. Well-newborn nursery
     7. Pediatrics inpatient unit
     8. Pediatric department/ward
     9. Other (please specify):
  3. What type of NICU does your hospital have?
     1. Intermediate (Level I or II)
     2. Community (Level III)
     3. Regional (Level IV)
     4. No NICU in hospital
     5. Other (please describe):
     6. Unsure
  4. What level of NAS does your hospital manage?
     1. We manage all NAS (mild to severe)
     2. We manage milder cases and transfer severe cases
     3. We transfer all NAS cases
     4. Other (please explain):
     5. Unsure
  5. In the past 6 months, approximately how many infants with NAS related to opioid exposure has your hospital cared for?
  6. During the past year, about how frequent were maternal-fetal opioid-related exposures at your hospital?
     1. Often (1 in 10 patients, or more often)
     2. Sometimes (between 1 in 10 patients and 1 in 100 patients)
     3. Seldom (1 in 100 patients, or less often)
     4. Never
     5. Unsure
  7. During the past year, how frequently were infants observed for NAS related to opioid exposure managed **using non pharmacological techniques,** such as rooming in, breastfeeding, reducing stimulation, etc.?
     1. Always
     2. Usually
     3. About Half the Time
     4. Seldom
     5. Never
     6. Unsure
  8. During the past year, how frequently were infants observed for NAS related to opioid exposure managed **pharmacologically for NAS** (e.g. morphine)?
     1. Always
     2. Usually
     3. About Half the Time
     4. Seldom
     5. Never
     6. Unsure
  9. Which of the following methods does the hospital use to determine infants’ substance exposure? [Source: Bogen 2017; Expanded response list based on Hudak AAP 2012 guidance]

|  | Yes | No | Unsure |
| --- | --- | --- | --- |
| Formal screening tool to obtain maternal history of drug use |  |  |  |
| Informal history to obtain maternal history of drug use |  |  |  |
| Maternal toxicology screening of urine, blood, or hair |  |  |  |
| Meconium |  |  |  |
| Infant urine |  |  |  |
| Infant blood |  |  |  |
| Umbilical cord |  |  |  |
| Infant hair |  |  |  |
| Other (please describe): |  |  |  |

- 1. Does your hospital have a **written protocol** in place to determine infants’ substance exposure? [Adapted from Bogen 2017]
     1. Yes
     2. No
     3. Unsure
  2. Which infants receive a toxicology screening (e.g. urine or other lab test) for substance exposure?

|  | Yes | No | Unsure |
| --- | --- | --- | --- |
| At risk newborns |  |  |  |
| All newborns in the NICU |  |  |  |
| Other (please describe): |  |  |  |

- 1. Does your hospital have **written protocols** for hospital management of NAS?
     1. Yes
     2. No
     3. Unsure
  2. What kinds of **written protocols** does your hospital have regarding hospital management NAS? Please skip if question is not applicable to your hospital. [Source: Adapted from Bogen 2017]

|  | Yes | No | Unsure |
| --- | --- | --- | --- |
| Nursing management |  |  |  |
| Non-pharmacologic management such as rooming in, swaddling, decreasing stimulation, etc. |  |  |  |
| Breastfeeding |  |  |  |
| Initiation of pharmacologic management |  |  |  |
| Dose escalation of pharmacologic management |  |  |  |
| Weaning of pharmacologic management |  |  |  |
| Discharge planning |  |  |  |
| Transfers to other hospitals |  |  |  |
| Other (please describe): |  |  |  |

- 1. Is training related to NAS (such as awareness and education) included in clinical staff training at your hospital? Include training given to nurses, physicians and other healthcare workers.
     1. Yes
     2. No
     3. Unsure
  2. When is NAS-related training typically offered to clinical staff? (Select all that apply). Please skip if question is not applicable to your hospital.
     1. At orientation
     2. During a relevant case
     3. As CME credits throughout the year
     4. At meetings or seminars throughout the year
     5. Other:
     6. Unsure
  3. What type of education/training related to NAS is included in clinical staff training at your hospital? Please skip if question is not applicable to your hospital. [Source: Adapted from Marcellus 2012, Bogen 2017]

|  | Yes | No | Unsure |
| --- | --- | --- | --- |
| Care of substance-exposed infants |  |  |  |
| Standardization of NAS scoring or assessment |  |  |  |
| Training on hospital NAS protocols |  |  |  |
| Other (please describe): |  |  |  |

- 1. In general, how are signs and symptoms of NAS assessed? [Source: Bogen 2017]

|  | Yes | No | Unsure |
| --- | --- | --- | --- |
| Formal scoring system (e.g. Finnegan tool) |  |  |  |
| Clinical exams or assessments, not a formal scoring system |  |  |  |
| Other, please describe: |  |  |  |

- 1. Which NAS assessments are usually used? Please skip if question is not applicable to your hospital. [Source: Bogen 2017; edited response option list based on McQueen NEJM 2016] [ [Options randomized]

|  | Yes | No | Unsure |
| --- | --- | --- | --- |
| Finnegan NAS tool |  |  |  |
| Finnegan NAS Scale Short Form |  |  |  |
| Other modified Finnegan NAS tool |  |  |  |
| Lipsitz tool |  |  |  |
| Mother NAS Scale |  |  |  |
| Eat, Sleep, Console (ESC) assessment |  |  |  |
| Neonatal Narcotic Withdrawal Index (NNWI) |  |  |  |
| Other functional assessment, explain: |  |  |  |

- 1. How useful would it be to receive additional guidance to raise the quality of NAS care related to opioid exposure at your hospital?
     1. Very useful
     2. Somewhat useful
     3. Not too useful
     4. Not at all useful
  2. For infants being observed or treated for NAS related to opioid exposure, approximately what is the average length of the infant’s total hospital stay (including NICU care)?

# of days (please enter)___________

- 1. Does the hospital offer **rooming-in** to mothers of infants being observed or treated for NAS? Please skip if question is not applicable to your hospital. [Source: Boucher 2017; edited]
     1. Yes
     2. No
     3. Unsure
  2. **Where** does the hospital offer **rooming-in** for mothers of infants being observed or treated for NAS**?** Please skip if question is not applicable to your hospital. [Source: Boucher 2017; edited] [Options randomized]

|  | Yes | No | Unsure |
| --- | --- | --- | --- |
| Well-newborn nursery |  |  |  |
| NICU |  |  |  |
| PICU |  |  |  |
| Pediatrics inpatient unit |  |  |  |
| Pediatric department/ward |  |  |  |
| Maternity/post-partum unit |  |  |  |
| Some other placement (please describe): |  |  |  |

- 1. Is early **skin-skin** contact with the birth mother prioritized for infants being observed or treated for NAS? Please skip if question is not applicable to your hospital. [Source: Adapted from NeoQIC]
     1. Always
     2. Usually
     3. About Half the Time
     4. Seldom
     5. Never
     6. Unsure
     7. Other, please specify:_____
  2. Please indicate the first-line therapy for NAS related to opioid exposure (please skip if question is not applicable to your hospital): [NEW]
     1. Nonpharmacologic interventions (e.g. quiet environment, swaddling)
     2. Pharmacologic (e.g. morphine) intervention
     3. Other, please describe:
     4. Unsure
  3. Which non-pharmacologic **environmental** interventions does your hospital routinely use for the treatment of NAS symptoms related to opioid exposure? (Select all that apply). Please skip if question is not applicable to your hospital. [Source: Bogen 2017; expanded response list based on recent literature including BWH guidelines] [Options randomized]
     1. Low level lighting
     2. Cover isolette/crib
     3. Music therapy
     4. Quiet environment
     5. Swaddling
     6. Sleep positioning
     7. Warm blanket
     8. Rooming-in
     9. Other (please describe):
  4. What **other** non-pharmacologic interventions does your hospital routinely use for the treatment of NAS symptoms related to opioid exposure? (Select all that apply). Please skip if question is not applicable to your hospital. [Source: Bogen 2017; expanded response list based on recent literature including BWH guidelines] [Options randomized]
     1. Skin-to-skin care
     2. Breastfeeding
     3. Massage or rubbing
     4. Holding
     5. Gentle rocking
     6. Gentle containment/pressure
     7. Slow infant handling
     8. Acupuncture
     9. Delaying circumcision
     10. Empowering messages to caregiver
     11. Volunteer “cuddler” program
     12. Other (please describe):
  5. In your hospital, what proportion of infants with NAS related to opioid exposure are treated with medications? Please skip if question is not applicable to your hospital. [NEW]
     1. All
     2. Most
     3. About half
     4. Some
     5. Rarely
     6. None
  6. Please select the most common first-line and second-line medications used to treat NAS related to opioid exposure in your hospital. (Select all that apply). Please skip if question is not applicable to your hospital.

| Pharmacologic agent | First-line therapy | Second-line therapy | Unsure |
| --- | --- | --- | --- |
| Morphine (oral) |  |  |  |
| Morphine (IV) |  |  |  |
| Methadone |  |  |  |
| Buprenorphine |  |  |  |
| Clonidine |  |  |  |
| Diazepam |  |  |  |
| Phenobarbital |  |  |  |
| Paregoric (e.g. camphorated tincture of opium) |  |  |  |
| Tincture of Opium (e.g. Laudanum, deodorized opium tincture) |  |  |  |
| Other: |  |  |  |

- 1. In your hospital, how often are infants with opioid-related NAS who are **not pharmacologically managed** for NAS kept with their mother for the entire duration of the infant’s stay if they are medically stable? Please skip if question is not applicable to your hospital. [Source: Bogen 2017]
     1. Always
     2. Usually
     3. About Half the Time
     4. Seldom
     5. Never
     6. Unsure
  2. In your hospital, how often are infants with opioid-related NAS who are being **pharmacologically managed** for NAS kept with their mother for the entire duration of the infant’s stay if they are medically stable? Please skip if question is not applicable to your hospital. [Source: Bogen 2017]
     1. Always
     2. Usually
     3. About Half the Time
     4. Seldom
     5. Never
     6. Unsure
  3. Thinking of **all infants** born at the hospital, how often are infants breastfed during their hospital stay? [Adapted Bogen 2017]
     1. Always
     2. Usually
     3. About Half the Time
     4. Seldom
     5. Never
     6. Unsure
  4. How often are infants under observation for or treated **for NAS** related to opioid exposure breastfed during their hospital stay? Please skip if question is not applicable to your hospital. [Adapted Bogen 2017]
     1. Always
     2. Usually
     3. About Half the Time
     4. Seldom
     5. Never
     6. Unsure
  5. If the mother of an infant observed or treated **for NAS** desires to breastfeed and has no contraindications (e.g. no infectious disease), she is **discouraged** from breastfeeding if there is (please skip if question is not applicable to your hospital):

|  | Yes | No | Unsure |
| --- | --- | --- | --- |
| Any illicit drug use related to opiates |  |  |  |
| Any illicit drug use related to methamphetamines |  |  |  |
| Any illicit drug use related to cocaine |  |  |  |
| Drug use limited to marijuana only |  |  |  |
| Alcohol use or alcohol use disorder |  |  |  |
| Methadone or buprenorphine treatment for opioid use disorder |  |  |  |
| Enrollment in a stable drug or alcohol treatment program |  |  |  |
| Other specific illicit drug use (please describe): |  |  |  |
| Not discouraged from breastfeeding for any of the above issues |  |  |  |

- 1. Of all the infants with NAS related to opioid exposure who are treated with medications, how often are they discharged home on medications? Please skip if question is not applicable to your hospital. [Source: Bogen 2017; edited for readability and responses revised]
     1. Always
     2. Usually
     3. About Half the Time
     4. Seldom
     5. Never
     6. Unsure
  2. What kind of post-discharge follow-up is provided to the parent, guardian, or caretaker of infants under observation for or being treated for NAS related to opioid exposure? Please skip if question is not applicable to your hospital. [Options randomized]

|  | Yes | No | Unsure |
| --- | --- | --- | --- |
| Referral for maternal primary care physician visits |  |  |  |
| Scheduling of maternal primary care physician visit(s) |  |  |  |
| Referral for infant primary care visit |  |  |  |
| Scheduling of pediatrician visit(s) |  |  |  |
| Home nursing visit |  |  |  |
| Referral to a specialized program for NAS or high risk infants |  |  |  |
| Other (please describe): |  |  |  |

- 1. Are you familiar with home visiting services in your community. Please skip if question is not applicable to your hospital?
     1. Yes (please list these services):
     2. No
     3. Unsure
  2. Which of the following would have the most impact on improving care for infants observed or treated for NAS at your hospital? **Select the five most important**. [NEW] [Options randomized]
     1. More patient engagement/interest
     2. More clinician engagement/interest
     3. Providing a higher level of care (e.g. a higher-level nursery)
     4. More clinician education
     5. Creation of guidelines/protocols or best practices
     6. Promotion of guidelines/protocols or best practices
     7. More integration between different types of care and care providers
     8. More transparency in current practices
     9. Addressing clinician stigma related to perinatal substance use
     10. Addressing patient stigma related to perinatal substance use
     11. More hospital internal/leadership support
     12. Improving payment systems
     13. More prenatal counseling
     14. More follow-up with infants and parents/caregivers
     15. Greater staff appreciation for nonpharmacologic treatments
     16. Greater parents/caregiver appreciation for nonpharmacologic treatments
     17. More culturally appropriate care practices
     18. Clinician training from ambulatory addiction medicine providers on best practices
     19. More social workers and peer advocates to assist in maternal recovery
     20. Improve staff knowledge of community-based supports and services for moms and babies
     21. Other, please describe: [TEXT BOX]
     22. Unsure
  3. If you would like to receive a $20 Amazon gift card, please enter the email address of where we can send the gift.

**Sources**

Bogen, D. L., Whalen, B. L., Kair, L. R., Vining, M., & King, B. A. (2017). Wide Variation Found in Care of Opioid-Exposed Newborns. *Academic Pediatrics*, *17*(4), 374–380. https://doi.org/10.1016/j.acap.2016.10.003

Comittee on Fetus and Newborn. (2012). *Levels of Neonatal Care* (Policy Statement). American Academy of Pediatrics. Retrieved from http://pediatrics.aappublications.org/content/130/3/587

Data Collection Standards for Race, Ethnicity, Sex, Primary Language, and Disability Status. (2013, September 6). Retrieved January 8, 2018, from

Hudak, M. L., & Tan, R. C. (2012). Neonatal Drug Withdrawal. *Pediatrics*, *129*(2), e540. https://doi.org/10.1542/peds.2011-3212

Marcellus, L., Loutit, T., & Cross, S. (2015). A National Survey of the Nursing Care of Infants With Prenatal Substance Exposure in Canadian NICUs. *Advances in Neonatal Care : Official Journal of the National Association of Neonatal Nurses*, *15*(5), 336–344. https://doi.org/10.1097/ANC.0000000000000165

NeoQIC, & MPQC. (n.d.). Improving the Care of Opioid-Exposed Newborns and their Families: A PNQIN Initiative. Retrieved January 8, 2018, from https://www.neoqicma.org/substance-exposed-newborns

O’Grady, M. J., Hopewell, J., & White, M. J. (2009). Management of neonatal abstinence syndrome: a national survey and review of practice. *Archives of Disease in Childhood. Fetal and Neonatal Edition*, *94*(4), F249–252. https://doi.org/10.1136/adc.2008.152769

Patrick, S. W., Schumacher, R. E., Horbar, J. D., Buus-Frank, M. E., Edwards, E. M., Morrow, K. A., … Soll, R. F. (2016). Improving Care for Neonatal Abstinence Syndrome. *Pediatrics*, *137*(5). https://doi.org/10.1542/peds.2015-3835

SAMHSA. 2018. “Clinical Guidance for Treating Pregnant and Parenting Women With Opioid Use Disorder and Their Infants.” https://store.samhsa.gov/product/Clinical-Guidance-for-Treating-Pregnant-and-Parenting-Women-With-Opioid-Use-Disorder-and-Their-Infants/SMA18-5054.

UCSF Northern California Neonatal Consortium. (6/14). *Consensus Guidelines for Management of Neonatal Abstinence Syndrome & Drug-Exposed Infants*. Retrieved from https://www.ucsfbenioffchildrens.org/pdf/abstinence_full_consensus_guideline.pdf

Wexelblatt, S. L., McAllister, J. M., Nathan, A. T., & Hall, E. S. (2017). Opioid Neonatal Abstinence Syndrome: An Overview. *Clinical Pharmacology and Therapeutics*. https://doi.org/10.1002/cpt.958
